# Associations of Screen Time with Bedtime, Sleep, and Physical Activity Among School-Aged Children in Riyadh: A Cross-Sectional Study

**DOI:** 10.3390/jcm15218275

**Published:** 2026-10-23

**Authors:** Ahmed S. Alhowimel, Aqeel M. Alenazi, Bader A. Alqahtani, Mazyad A. Alotaibi, Norah A. Alhwoaimel, Abdullah H. AlMuhaya

**Affiliations:** 1 Department of Health and Rehabilitation Sciences, Prince Sattam Bin Abdulaziz University, Alkharj 11942, Saudi Arabia; a.alhowimel@psau.edu.sa (A.S.A.); aqeelalenazi.pt@gmail.com (A.M.A.); dralqahtaniba@gmail.com (B.A.A.); maz.alotaibi@psau.edu.sa (M.A.A.); n.alhwoaimel@psau.edu.sa (N.A.A.); 2 Joint Clinics, Medical Rehabilitation Center, Riyadh 13515, Saudi Arabia

**Keywords:** screen time, bedtime, sleep quality, sleep duration, physical activity, school-aged children, Saudi Arabia

## Abstract

**Background/Objectives:** Evidence on screen use and sleep among younger children in the Middle East is limited. This study examined associations of screen time with bedtime, sleep duration, sleep quality, and physical activity among school-aged children in Riyadh, Saudi Arabia. **Methods:** In this cross-sectional study (September–October 2025), parents of 1208 children aged 4–15 years attending one private school completed an online survey. Associations were examined using chi-square tests, Goodman–Kruskal Gamma, and ordinal and binary logistic regression adjusted for age and sex. **Results:** The proportion going to bed at or after 22:00 rose from 7.4% among those with <1 h/day of screen time to 32.9% among those with >3 h/day, and the corresponding proportions not waking refreshed were 20.1% and 35.1%. Compared with >3 h/day, 1–2 h/day was associated with lower odds of late bedtime (odds ratio [OR] = 0.37, 95% confidence interval [CI] 0.23–0.62) and non-restorative sleep (OR = 0.48, 95% CI 0.32–0.72), and estimates were stable across sensitivity analyses. The unadjusted association with shorter sleep (Gamma = −0.336) was attenuated after adjustment for age, and screen time was not inversely associated with physical activity. **Conclusions:** Higher screen time was associated with later bedtimes and non-restorative sleep, but not with lower physical activity, suggesting that sleep-focused guidance on evening screen use merits evaluation in longitudinal and intervention studies.

## 1. Introduction

Digital media use has become an integral part of how children learn, communicate, and play [1]. Children now use a wide range of devices, including smartphones, tablets, televisions, and gaming systems, from early childhood [2], and a growing proportion of school-aged children exceed recommended screen-time limits [1]. Because devices are available throughout the day and night, screen use may displace time otherwise allocated to sleep, physical activity, and family interaction [3,4]. Measuring screen time has also become more difficult because of task switching, background media, and simultaneous use of multiple devices [2].

Higher screen time has been consistently associated with poorer sleep in children and adolescents. A systematic review found adverse associations, primarily delayed sleep timing and shorter sleep duration, in most studies [5], and a meta-analysis linked bedtime access to portable media devices with inadequate sleep quantity, poor sleep quality, and daytime sleepiness [6]; cross-sectional and longitudinal studies report similar associations with sleep duration [7,8]. Bedtime displacement, whereby screen use delays going to bed, is one of the most commonly proposed mechanisms, particularly for evening use [9]. Screen time has also been linked to waking unrefreshed and other indicators of poor sleep quality, even when sleep duration is within the recommended range [10,11], possibly through light-induced melatonin suppression, pre-sleep arousal [3,12], and effects on developing arousal-regulation systems [13].

Evidence for physical activity is less consistent. Higher screen exposure is generally associated with more sedentary time, less moderate-to-vigorous physical activity, and cardiometabolic risk [14,15,16,17], but organized sport and school physical education may offset screen-based habits [18], and several studies report weak or non-monotonic associations [19]. Cross-sectional designs may also miss compensatory patterns, such as children who spend many hours on screens yet participate in organized sport [20].

Despite the abundance of literature on pediatric screen time, evidence from the Middle East remains limited, particularly for younger school-aged children. In Saudi Arabia, most available data describe adolescents: a large multicenter survey reported that 84% of male and 91% of female Saudi adolescents exceeded 2 h of daily screen time [21], and a recent study reported mean screen times of approximately 4 h/day among Saudi male adolescents, regardless of sports participation [22]. Data on younger children are fewer but point in the same direction: among 1051 elementary school children in Riyadh aged approximately 6–13 years, mean screen time was 3.2 h/day, more than 71% did not obtain sufficient nighttime sleep, and screen time above 2 h/day was associated with lower odds of sufficient sleep [23]; a national survey of children aged 6–12 years conducted during the COVID-19 pandemic found that only 15% met the recreational screen-time recommendation [24]; and among children aged 2–14 years in Riyadh, bedtime resistance (70.3%) and sleep-onset delay (58.1%) were common and were associated with screen time [25]. Although most Saudi parents are aware of the potential effects of electronic devices on children’s sleep, many report difficulty limiting device use [26]. Other regional studies have focused on university students [27], and few studies have examined bedtime, sleep quality, and physical activity together in children from the early school years onward. Rapid urbanization, widespread household digital access, and climatic conditions that limit outdoor activity may shape children’s screen, sleep, and activity behaviors differently in this setting. By including children from kindergarten through middle school in Riyadh, the present study addresses both the regional and the age-related gaps in this evidence. Therefore, this study aimed to examine the associations of daily screen time with bedtime, sleep duration, sleep quality, and physical activity among school-aged children in Riyadh, Saudi Arabia. We tested three directional hypotheses: that higher screen time would be associated with (i) later school-night bedtimes, (ii) shorter sleep duration, and (iii) poorer sleep quality, indicated by not waking refreshed. Given the inconsistent evidence summarized above, the association with physical activity was examined as an exploratory, non-directional question.

## 2. Materials and Methods

### 2.1. Study Design and Setting

This cross-sectional, school-based study was conducted in Riyadh, Saudi Arabia, between September and October 2025 using an online parent-reported survey. The study was implemented in one private school with two branches. The survey was administered as part of a larger school-based scoliosis screening program, in which parents completed the online survey before their children were screened at school. The study is reported in accordance with the Strengthening the Reporting of Observational Studies in Epidemiology (STROBE) guidelines [28].

### 2.2. Participants, Recruitment, and Eligibility Criteria

Eligible participants were children enrolled from kindergarten level 3 through Grade 9 (approximately 4–15 years of age). A parent or legal guardian completed the survey on behalf of the child. The school was recruited using convenience sampling based on administrative access and willingness to participate.

**Figure 1:**
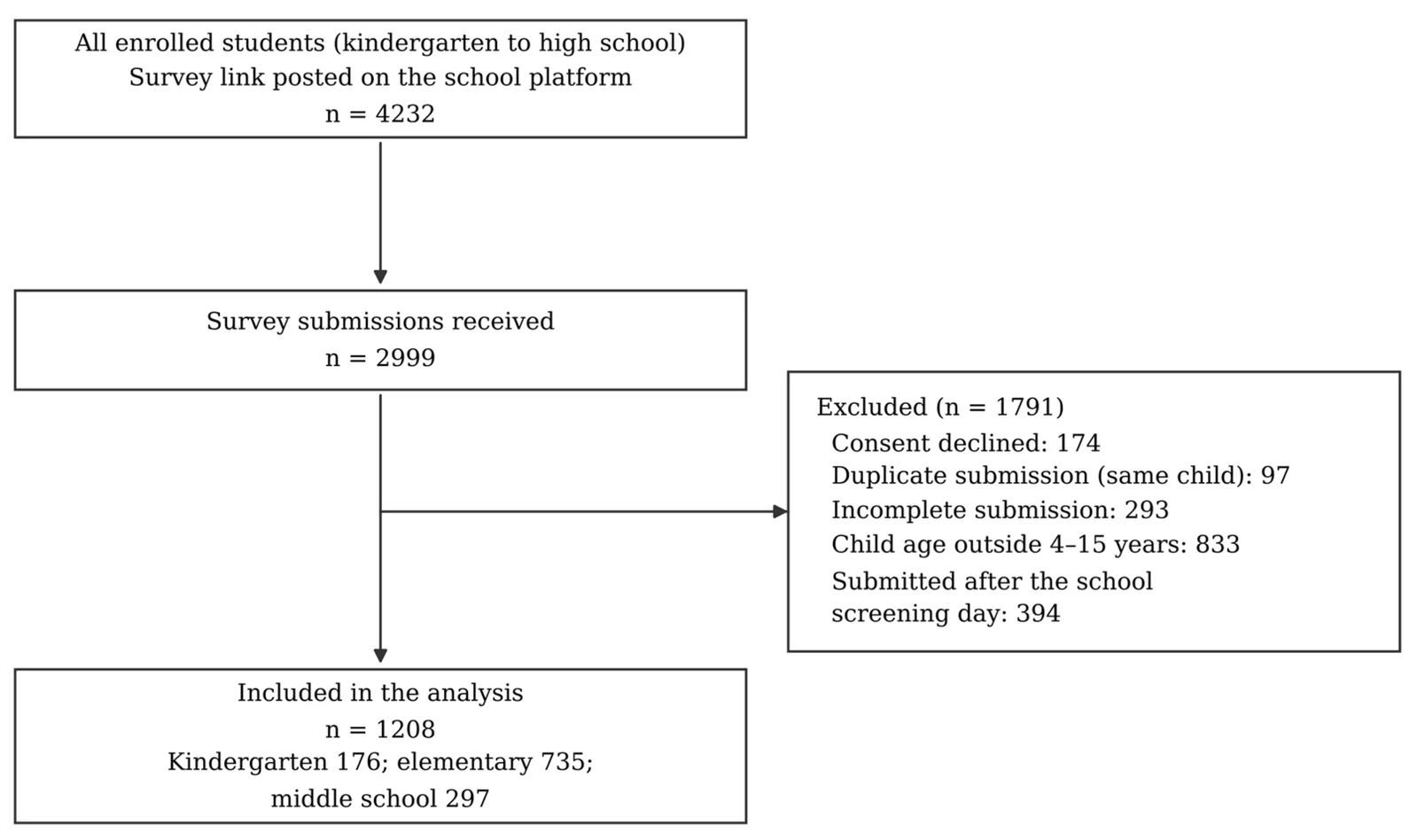
Flow of participants. Submissions were excluded sequentially for the reasons shown.

The school administration posted the survey link on the school’s official online platform, where it was accessible to the parents or legal guardians of all 4232 students enrolled from kindergarten to high school. The invitation message accompanying the link stated that the survey was part of a research study. Parents could complete the survey once for each of their children. A total of 2999 submissions were received, of which 1208 met the criteria for a valid response and were included in the analysis (Figure 1). Because the platform did not record which families viewed the link and the denominator included students outside the eligible age range, a formal response rate could not be calculated; the approximate participation proportion was 28.5% (1208/4232), and 40.3% of the submissions received (1208/2999) were valid. School branch was recorded in the survey but was not retained in the analyzed dataset, so branch-specific participation could not be calculated, and no information was available on the characteristics of non-responding families. Participation was voluntary, and electronic informed consent was required before survey access. The target sample of 1000–1200 children was based on the available distribution pool and exceeded the minimum required for the planned logistic regression models, based on the rule of at least 10 outcome events per predictor term.

Inclusion criteria were enrollment in the participating school, a child age of 4–15 years, and completion of the survey by a parent or legal guardian before the school screening day. The survey items on screen time, sleep, and physical activity were mandatory, whereas the child’s demographic and anthropometric details (e.g., age, grade, height, and weight) were optional. A valid response was defined as a submission with parental consent that was not a duplicate, had complete demographic and anthropometric information, referred to a child aged 4–15 years, and was received before the school screening day.

### 2.3. Duplicate Prevention and Data Integrity

Submissions were excluded sequentially for declined consent (*n* = 174), duplicate submission for the same child (*n* = 97), incomplete demographic or anthropometric information (*n* = 293), child age outside 4–15 years (*n* = 833), and submission after the school screening day (*n* = 394) (Figure 1). Duplicates were identified using the child’s name and the parent’s phone number as identifiers; when the same child appeared more than once, only one response was retained. Submissions with the same parent phone number but different children were retained as siblings; 42 children (3.5%) from 21 households were siblings. Submissions received after the screening day were excluded because the survey link remained open on the school platform, these children had not been screened, and a parent awareness workshop held after the screening could have influenced responses. Free-text categorical responses (e.g., sex) were standardized for letter case before analysis. Body mass index (BMI) was calculated from parent-reported height and weight, and five biologically implausible values (BMI below 10 or above 40 kg/m^2^) were set to missing. Data cleaning was completed before statistical analysis.

### 2.4. Variables and Measures

All measures were reported by parents or legal guardians using predefined response categories. The exposure, screen time, was assessed as the child’s average daily time spent on screens (television, phone, and computer) outside school and was categorized as <1 h, 1–2 h, 2–3 h, and >3 h per day. This single item captured total screen use outside school hours and did not distinguish recreational from educational use, device type, content, or the timing of use relative to bedtime. The screen-time and sleep-duration items asked about the child’s usual (average) behavior without a specified recall period and did not distinguish school days from weekends, whereas the bedtime item referred specifically to school days. The screen-time categories were chosen to bracket the commonly recommended limit of 2 h/day of recreational screen time for school-aged children, allowing comparison with guideline-based thresholds used in previous studies [21,24].

Four outcomes were examined. Bedtime on school days was reported using the options 18:00–19:00, 20:00–21:00, 22:00, and after 22:00; five free-text responses (between 19:00 and 21:30) were assigned to the nearest category, yielding four ordered categories (before 20:00, 20:00–21:00, around 22:00, and after 22:00). Late bedtime was defined as going to bed at or after 22:00. Sleep duration was assessed as average nightly sleep (<7 h, 7–8 h, 9–10 h, and >10 h). Sleep quality was assessed with a single item, “Does your child usually wake up feeling refreshed in the morning?” (yes/no), and a “no” response was defined as non-restorative sleep. Physical activity was assessed as the average daily time spent in sports, outdoor play, or walking (<30 min, 30–60 min, 1–2 h, and >2 h per day). Because the sleep-duration options did not include 8–9 h and the bedtime options did not include 19:00–20:00 or 21:00–22:00, parents of children whose usual values fell within these intervals had to select an adjacent category.

Covariates included child age (years), sex, school level (kindergarten, elementary, and middle school), BMI, and any parent-reported chronic health condition (free-text item, coded as any vs. none). Daily time spent sitting at school and regular breaks from sitting (every 30–60 min; yes/no) were recorded descriptively. Throughout the manuscript, “screen time” refers to out-of-school screen time, “bedtime” to school-night bedtime, “sleep duration” to usual nightly sleep duration, and “non-restorative sleep” to not usually waking refreshed.

### 2.5. Survey Instrument

A custom-designed online survey was developed using Google Forms (Google LLC, Mountain View, CA, USA) to assess lifestyle behaviors relevant to school-aged children. Survey items and response categories were informed by prior pediatric research on screen time, sleep, and physical activity to support content relevance and face validity. The survey was reviewed for clarity and appropriateness for parent-reported responses before dissemination. It was administered bilingually, with each item presented in both Arabic and English, and the English version of the items analyzed in this study is provided in Appendix A. Before distribution, the survey was pilot-tested with a small group of parents, who found the items clear and easy to understand. Items were selected to be brief and observable by parents; the sleep-quality item was chosen as a practical indicator of restorative sleep and is conceptually similar to the morning-waking items of the daytime sleepiness domain of the Children’s Sleep Habits Questionnaire [29]. However, no formal assessment of content validity, construct validity, or test–retest reliability was undertaken; the psychometric properties of the items, including the single-item sleep-quality measure, have therefore not been established in this population.

### 2.6. Ethical Considerations

The present study is a secondary analysis of existing, de-identified survey data collected in September–October 2025 within a larger school-based scoliosis screening program, and it was approved by the Research Ethics Committee, Department of Health and Rehabilitation Sciences, Prince Sattam bin Abdulaziz University (RHPT/026/020; 2 September 2026). No additional data were collected for the present analysis. The participating school administration granted permission. The invitation message accompanying the survey link stated that the survey was part of a research study, and parents or legal guardians provided electronic informed consent before completing the survey. Parent phone numbers and child names were collected solely to prevent duplicate responses and were removed following data cleaning. All analyses were conducted on a de-identified dataset in accordance with the Declaration of Helsinki.

### 2.7. Statistical Analysis

**Figure 2:**
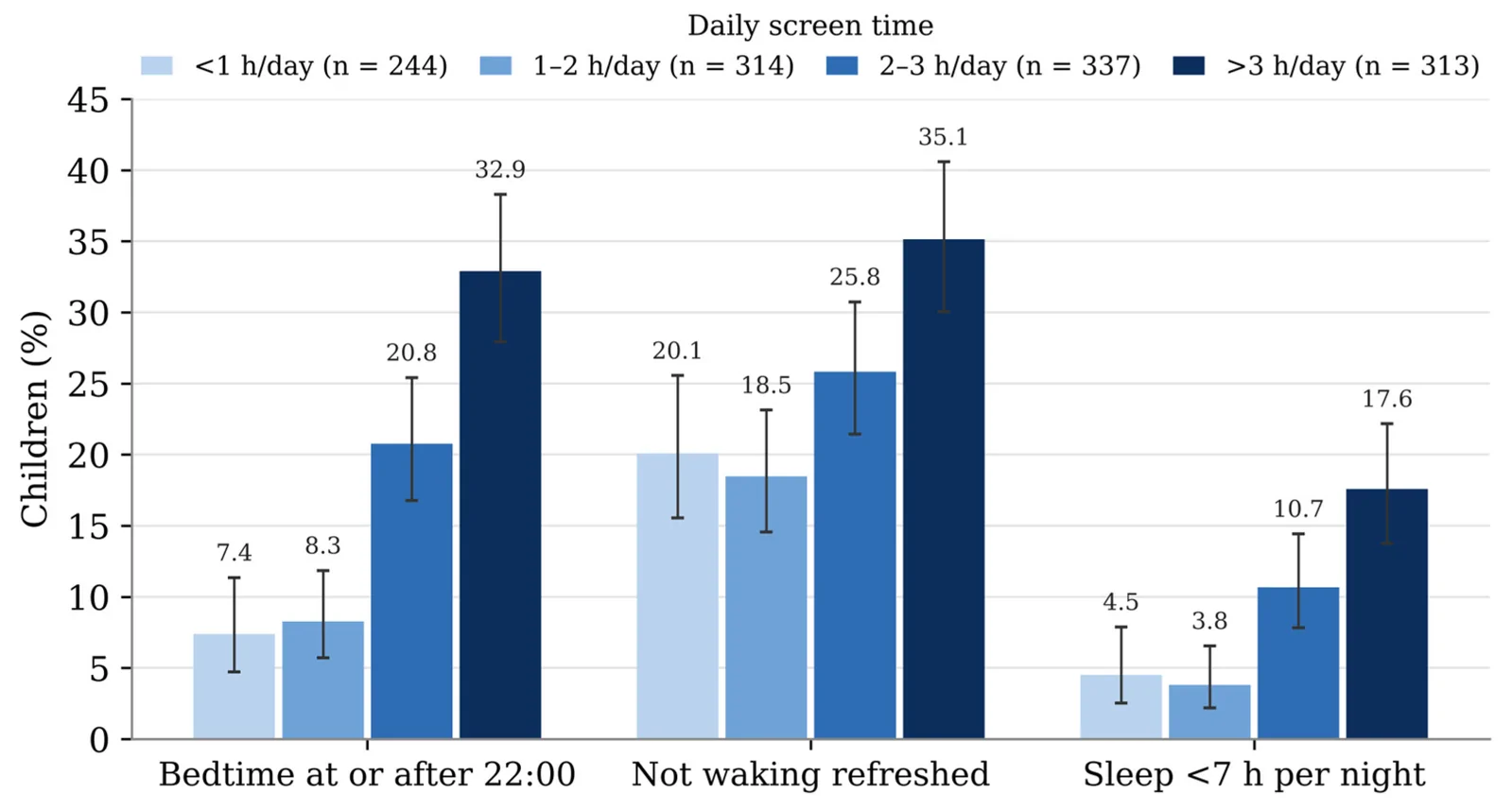
Proportion of children going to bed at or after 22:00, not waking refreshed, and sleeping <7 h per night, by daily screen time (*n* = 1208). Values above bars are percentages within each screen-time category. Error bars indicate 95% confidence intervals.

Analyses were conducted using JASP (version 0.95; University of Amsterdam, Amsterdam, The Netherlands) and Python (version 3.13; Python Software Foundation, Wilmington, DE, USA) with the pandas (version 3.0), SciPy (version 1.18), statsmodels (version 0.15), and matplotlib (version 3.11) libraries, and estimates were cross-checked between the two environments. Continuous variables were summarized as means ± standard deviations (SD) and categorical variables as frequencies and percentages. Figure 1 and Figure 2 were generated in Python using matplotlib. During the preparation of this study, the authors used Claude (Claude Opus 4.7 and Claude Opus 5.5; Anthropic, San Francisco, CA, USA) for the purposes of writing and checking the Python analysis code; all code was reviewed and run by the authors, and the main estimates were cross-checked against JASP output.

Unadjusted associations between screen time and each outcome were examined using chi-square tests, with Cramér’s V as the effect size. The direction and strength of ordered (dose–response) associations were summarized using Goodman–Kruskal Gamma with 95% confidence intervals (CIs) and Kendall’s τ_b_.

Adjusted associations were estimated using regression models that included age and sex as covariates. Late bedtime and non-restorative sleep were modeled using binary logistic regression. The proportional odds assumption was assessed for each ordinal outcome using the Brant test and by comparing ORs across cumulative binary splits (Table S1). The assumption was violated for bedtime (omnibus χ^2^(6) = 15.17, *p* = 0.019; screen time χ^2^(2) = 7.09, *p* = 0.029), which was therefore analyzed as a binary outcome (late vs. not late). For sleep duration, the Brant test did not indicate a violation (χ^2^(6) = 8.61, *p* = 0.197), but the screen-time ORs declined across the cumulative splits (0.71, 0.88, and 0.98); sleep duration was therefore analyzed with category-specific binary models for short sleep (<7 h vs. ≥7 h) and longer sleep (≥9 h vs. ≤8 h) rather than a single ordinal model. Physical activity was modeled using proportional odds (ordinal logistic) regression; because the omnibus Brant test was significant (*p* = 0.019), although the screen-time component was not (*p* = 0.071), split-specific estimates are also reported (Table S1). Screen time >3 h/day served as the reference category, and a linear trend was tested by entering screen time as an ordinal score (0–3). Because this score assumes a constant change in the log odds across categories, departure from linearity was assessed with LR tests comparing the categorical and linear-score models (2 df), and category-specific ORs are reported for all outcomes. Because age and school level were strongly correlated (r = 0.87), age was retained in the main models and school level was examined in sensitivity analyses.

Sensitivity analyses for late bedtime and non-restorative sleep (i) replaced age with school level, (ii) additionally adjusted for chronic health conditions and BMI, and (iii) excluded children with any reported health condition. To explore whether bedtime accounted for part of the association with sleep quality, bedtime was added to the non-restorative sleep model. Associations were also examined separately by school level, and screen time × school level interactions were tested using likelihood ratio (LR) tests. Additional sensitivity analyses (i) collapsed the 7–8 h and 9–10 h sleep-duration categories (<7, 7–10, and >10 h), (ii) adjusted all outcome models for age, sex, BMI, and chronic health conditions, and (iii) estimated by simulation (1000 replicates) the power of the kindergarten subgroup to detect associations of the magnitude observed among elementary school children. To account for possible correlation among children in the same grade, the screen-time trend models were re-estimated with cluster-robust standard errors using school grade as the cluster (10 grades), with CIs and *p* values based on the t distribution with 9 degrees of freedom (Table S5). Household and school branch identifiers were not retained in the analyzed dataset, so clustering at these levels could not be modeled. Statistical significance was set at *p* < 0.05.

Sleep duration and sleep quality were designated as the primary outcomes in the study protocol; bedtime was a secondary outcome, and the physical activity, subgroup, interaction, sensitivity, and bedtime-adjusted analyses were considered exploratory. To account for multiple testing, the Benjamini–Hochberg false discovery rate (FDR) procedure was applied to the screen-time trend tests across outcomes and to the 15 category-specific screen-time contrasts (Table S2).

## 3. Results

### 3.1. Participant Characteristics

Of the 2999 submissions received, 1208 met the criteria for a valid response and were included in the analysis (Figure 1; mean age 9.1 ± 2.8 years; 57.5% female). Most attended elementary school (60.8%), followed by middle school (24.6%) and kindergarten (14.6%). More than half (53.8%) had ≥2 h/day of screen time, and 20.2% had <1 h/day. Most children went to bed between 20:00 and 21:00 (63.4%), whereas 18.0% went to bed at or after 22:00. Sleep duration was most frequently 7–8 h (45.0%) or 9–10 h (40.7%), and 9.4% slept <7 h. One quarter (25.2%) did not usually wake up refreshed. Nearly half (48.3%) engaged in <30 min of daily physical activity, and 76.0% sat for more than 6 h at school. A chronic health condition was reported for 15.9% of children, most commonly allergic or respiratory conditions (6.7%). Participant characteristics are presented in Table 1.

### 3.2. Unadjusted Associations Between Screen Time and Outcomes

Screen time was associated with all four outcomes (Table 2). The strongest ordered association was observed for bedtime (χ^2^(9) = 132.31, *p* < 0.001; Cramér’s V = 0.191; Gamma = 0.403, 95% CI 0.335–0.472): the proportion of children going to bed at or after 22:00 was 7.4%, 8.3%, 20.8%, and 32.9% across increasing screen-time categories (Figure 2). Higher screen time was also associated with shorter sleep duration (χ^2^(9) = 112.03, *p* < 0.001; V = 0.176; Gamma = −0.336, 95% CI −0.403 to −0.269; Kendall’s τ_b_ = −0.232, *p* < 0.001) and with not waking refreshed (χ^2^(3) = 27.44, *p* < 0.001; V = 0.151; Gamma = 0.237, 95% CI 0.142–0.333), which increased from 18.5 to 20.1% among children with ≤2 h/day to 35.1% among those with >3 h/day. Screen time was associated with physical activity categories at the nominal level (χ^2^(9) = 28.63, *p* < 0.001; V = 0.089), but the ordered association was negligible (Gamma = −0.064, 95% CI −0.136 to 0.008).

### 3.3. Adjusted Associations

After adjustment for age and sex (Table 3), children with 1–2 h/day and <1 h/day of screen time had lower odds of late bedtime (OR = 0.37, 95% CI 0.23–0.62; OR = 0.43, 95% CI 0.24–0.78) and of non-restorative sleep (OR = 0.48, 95% CI 0.32–0.72; OR = 0.56, 95% CI 0.36–0.86) than those with >3 h/day, and children with 2–3 h/day also had lower odds of non-restorative sleep (OR = 0.68, 95% CI 0.48–0.96). Each higher screen-time category was associated with 43% higher odds of late bedtime (OR = 1.43, 95% CI 1.19–1.71) and 26% higher odds of non-restorative sleep (OR = 1.26, 95% CI 1.09–1.45). There was no evidence of departure from linearity for late bedtime (LR test *p* = 0.246) or non-restorative sleep (*p* = 0.148).

Sleep duration was analyzed using category-specific models. Age was strongly associated with sleep duration (OR for sleeping ≥9 h per year = 0.74, 95% CI 0.70–0.78), and the association with screen time was attenuated after adjustment. Before adjustment, each higher screen-time category was associated with higher odds of sleeping <7 h (OR = 1.86, 95% CI 1.51–2.29) and lower odds of sleeping ≥9 h (OR = 0.63, 95% CI 0.57–0.71); after adjustment for age and sex, these estimates were 1.41 (95% CI 1.12–1.78) and 0.88 (95% CI 0.77–1.00), respectively (Table S3). Short sleep therefore remained associated with screen time after adjustment, with no evidence of departure from linearity (LR test *p* = 0.214). For sleeping ≥9 h, only the 1–2 h/day category differed significantly from >3 h/day (OR = 1.81, 95% CI 1.25–2.62); the pattern across categories was non-linear (LR test *p* < 0.001), and the trend estimate was not significant (*p* = 0.050).

In exploratory analyses, no inverse association was observed between screen time and physical activity after adjustment. Children with <1 h/day had lower odds of higher physical activity than those with >3 h/day (OR = 0.66, 95% CI 0.46–0.95), and the trend estimate was in the opposite direction to the displacement hypothesis (OR = 1.15, 95% CI 1.02–1.29). Boys were more physically active than girls (OR = 1.27, 95% CI 1.02–1.58). The trend estimate was unchanged after additional adjustment for BMI and chronic health conditions (OR = 1.15, 95% CI 1.03–1.29), and the split-specific estimates indicated that it reflected fewer children in the lowest activity categories rather than more children exceeding 2 h/day (Table S1).

### 3.4. Sensitivity and Subgroup Analyses

The associations of screen time with late bedtime and non-restorative sleep were materially unchanged when school level replaced age, after additional adjustment for chronic health conditions and BMI, and after excluding children with any reported health condition (Table 4). Later bedtime was itself strongly associated with non-restorative sleep in a graded manner (vs. before 20:00: 20:00–21:00, OR = 1.99, 95% CI 1.30–3.06; around 22:00, OR = 3.43, 95% CI 2.02–5.81; after 22:00, OR = 6.97, 95% CI 3.32–14.61). Adding bedtime to the non-restorative sleep model modestly attenuated the screen-time estimates (e.g., 1–2 h/day: OR = 0.55, 95% CI 0.37–0.82), which remained significant. Collapsing the 7–8 h and 9–10 h sleep-duration categories did not materially change the sleep-duration findings (trend OR for a longer sleep category = 0.80, 95% CI 0.67–0.96; 1–2 h/day vs. >3 h/day, OR = 2.15, 95% CI 1.28–3.61), and estimates for all outcomes were materially unchanged after adjustment for BMI and chronic health conditions (Table S3). After FDR correction, the screen-time trend remained significant for late bedtime (q < 0.001), sleep < 7 h (q = 0.007), non-restorative sleep (q = 0.004), and physical activity (q = 0.026), but not for sleep ≥ 9 h (q = 0.060); for the category-specific contrasts in Table 3, all associations with *p* < 0.05 remained significant except non-restorative sleep at 2–3 h/day (q = 0.055) and physical activity at <1 h/day (q = 0.053) (Table S2). When standard errors accounted for clustering by school grade, the screen-time trends remained significant for late bedtime (OR = 1.43, 95% CI 1.09–1.88), sleep < 7 h (OR = 1.41, 95% CI 1.17–1.71), and non-restorative sleep (OR = 1.26, 95% CI 1.04–1.53), whereas the trend for physical activity was attenuated (OR = 1.15, 95% CI 0.99–1.33) (Table S5).

In exploratory analyses stratified by school level, the associations were not statistically significant among kindergarten children (Gamma for bedtime = −0.013, 95% CI −0.237 to 0.211; for not waking refreshed = −0.002, 95% CI −0.300 to 0.296). Among elementary school children, screen time was associated with later bedtime (Gamma = 0.398, 95% CI 0.298–0.498), not waking refreshed (Gamma = 0.166, 95% CI 0.039–0.293), and shorter sleep (Gamma = −0.217, 95% CI −0.312 to −0.122). Among middle school children, the association with not waking refreshed was strongest (Gamma = 0.332, 95% CI 0.130–0.534). Screen time × school level interactions were not statistically significant for non-restorative sleep (LR test *p* = 0.080) or late bedtime (*p* = 0.505). However, the kindergarten subgroup included few events (12 late bedtimes and 26 children not waking refreshed), and simulated power to detect associations of the magnitude observed among elementary school children was low (28% for late bedtime and 17% for non-restorative sleep; Table S4), so the absence of significant associations in this subgroup does not indicate true effect modification.

## 4. Discussion

This study examined the associations of screen time with bedtime, sleep, and physical activity in a private-school sample of children in Riyadh and identified three principal findings. First, higher screen time was associated with later bedtimes and non-restorative sleep in a graded manner, independent of age and sex and robust across sensitivity analyses. Second, the apparent inverse association between screen time and sleep duration was largely explained by age and, after adjustment, was concentrated in the shortest sleep category. Third, screen time was not inversely associated with physical activity. Because the design was cross-sectional, these findings describe associations and do not establish that screen time causes later bedtimes or poorer sleep. Together, these findings suggest that, in this sample, screen exposure is more closely associated with sleep timing and sleep quality than with physical activity.

The association with bedtime was the strongest observed: approximately one in three children with more than 3 h/day of screen time went to bed at or after 22:00, compared with fewer than one in ten of those with less than 2 h/day. This is consistent with systematic reviews identifying delayed sleep timing as one of the most consistent correlates of screen use in children and adolescents [5,6] and is consistent with, although it cannot establish, bedtime displacement as a mechanism [9]. Later bedtime was, in turn, strongly associated with waking unrefreshed, and adding bedtime to the model modestly attenuated the association between screen time and non-restorative sleep. This pattern suggests that later bedtimes may account for part, but not all, of the association. Other pathways, such as pre-sleep cognitive and emotional arousal and evening light exposure [3,12], and effects on developing arousal-regulation systems [13], may also contribute. Because the data are cross-sectional, these pathways could not be formally tested as mediators. These pathways could be examined in longitudinal studies that record the timing, device, and content of evening screen use, assess sleep objectively (for example, with actigraphy) alongside validated sleep-quality measures, and test bedtime as a mediator using repeated measurements.

The association between screen time and non-restorative sleep was the most stable finding, with nearly identical estimates across all model specifications, including after excluding children with health conditions that may affect sleep. Similar associations between screen exposure and subjective sleep quality have been reported in other pediatric populations [10,11]. Although sleep quality was assessed using a single item, perceived morning refreshment is a meaningful and practical indicator of restorative sleep that parents can readily observe, although it has not been validated against standardized pediatric sleep instruments and may be less sensitive than multi-item measures.

In contrast, the strong unadjusted association with sleep duration was substantially attenuated after adjustment for age. Older children in this sample had more screen time and, consistent with age-specific sleep recommendations [30], shorter sleep, so failing to account for age would have overstated the association. After adjustment, higher screen time remained associated with sleeping less than 7 h, but estimates across categories were non-monotonic. Parental routines, school schedules, and culturally structured family bedtimes may partly preserve total sleep duration even when sleep timing shifts. These findings highlight the importance of age adjustment in pediatric screen–sleep research, particularly in samples spanning a wide age range. Because the sleep-duration options omitted 8–9 h, some misclassification between adjacent categories is likely; if unrelated to screen time, it would bias estimates toward the null, and collapsing these categories did not change the findings.

Screen time was not associated with lower physical activity, which aligns with studies reporting weak or non-monotonic associations between screen exposure and physical activity in children [19,20]. Participation in organized sport and compulsory physical education may provide a baseline level of activity that is relatively independent of leisure screen use [18], and prolonged periods of high temperature may encourage indoor activities, including screen use, without necessarily reducing structured physical activity [31]. The slightly higher activity observed with greater screen time after age adjustment was unexpected. It reflected movement out of the lowest activity categories, was unchanged after adjustment for BMI and chronic health conditions, and the category-specific contrast did not remain significant after FDR correction. Residual confounding is a plausible explanation: in a private-school sample, household income, parental education, and access to devices may be associated with both greater screen access and enrollment in organized sports or clubs, and differences in parental supervision of both behaviors could produce a similar pattern. These factors were not measured, and shared parent-report bias may also have contributed; the finding should therefore be interpreted cautiously and examined in studies with socioeconomic data and device-based activity measures.

The associations were not statistically significant among kindergarten children and were evident from elementary school onward; however, the interaction tests were not significant, and the kindergarten subgroup had limited power. Bedtimes and device access among younger children are likely to be more closely controlled by parents, whereas older children gain autonomy over both. The transition to elementary school may therefore be a relevant period for sleep-focused guidance, but this possibility is speculative and requires confirmation in adequately powered longitudinal studies.

These findings add to the limited evidence from Saudi Arabia, where high screen time among adolescents has been documented for more than a decade [21,22] and recent studies have reported high screen time and frequent sleep problems among younger children [23,24,25]; the present study extends this evidence by examining bedtime, sleep quality, and physical activity together in a large sample from the early school years onward. These findings are also consistent with international evidence that associations between screen use and sleep are generally stronger and more consistent than those with physical activity [4,32]. The high level of parental awareness but limited success in restricting device use reported in Saudi families [26] suggests that practical, specific guidance may be more useful than general advice to reduce screen time.

From a public health perspective, and recognizing that cross-sectional associations cannot show that reducing screen time would improve sleep, guidance may benefit from moving beyond general time-based limits toward specific sleep-focused strategies, such as ending screen use well before bedtime and maintaining consistent school-night bedtimes; these strategies are informed mainly by the broader literature on evening screen use [3,6,9] and were not directly tested in this study. Schools are a practical setting for such guidance, and health education could include evening device use and recognition of morning fatigue. Given the absence of an inverse association with physical activity, efforts to increase activity should focus on providing structured opportunities rather than assuming that reduced screen time will increase physical activity.

Strengths of this study include a large sample that includes younger children, the combination of ordinal and adjusted analyses rather than a single screen-time threshold, the inclusion of bedtime as an outcome, and multiple sensitivity analyses addressing age, school level, chronic health conditions, and BMI.

### Limitations

The cross-sectional design precludes causal inference; poorer sleep may promote screen use as well as result from it. The sampling approach may also have introduced selection bias. The study was conducted in a single private school using convenience sampling, with an approximate participation proportion of 28.5%, and no information was available on non-responders. Families who chose to respond may differ from non-responders in their screen-use practices and sleep routines, and private-school families are likely to have higher socioeconomic status than the general population. The findings therefore may not be generalizable to children in public schools, other socioeconomic groups, or the wider Riyadh or Saudi pediatric population, and prevalence estimates in particular should not be extrapolated.

All data were parent-reported, which may introduce recall and social desirability bias, particularly for older children who manage devices independently, the survey did not undergo formal psychometric validation, and its reliability was not assessed. The screen-time item did not distinguish recreational from educational use or capture the timing and content of screen use, which may be particularly relevant for sleep. Evening use is the most direct test of bedtime displacement, and its absence means that the associations may partly reflect daytime or educational screen use. Response categories were broad and, in some cases, non-contiguous: sleep duration options did not include 8–9 h, and bedtime options did not include 19:00–20:00 or 21:00–22:00, which may have introduced misclassification. If this misclassification was unrelated to screen time, it would bias estimates toward the null, whereas differential rounding by parents of children with high screen time (for example, reporting 22:00 for a 21:30 bedtime) could inflate the late-bedtime association. The short-sleep cut-point (<7 h) is not affected by the missing 8–9 h interval, and collapsing adjacent sleep-duration categories did not change the findings. The screen-time and sleep-duration items did not specify a recall period or distinguish school days from weekends, and the sleep measures capture only part of sleep health (timing, duration, and perceived restoration) and not sleep-onset latency, night awakenings, or daytime sleepiness. Sleep quality was assessed using a single item, physical activity was measured by duration rather than intensity, and BMI was based on parent-reported height and weight.

Important potential confounders, including household income, parental education, household composition, device ownership, bedroom media access, and parental monitoring of screen use, were not collected, so residual confounding cannot be excluded. School branch was recorded but not retained in the analyzed dataset, and household identifiers were removed after data cleaning; therefore, clustering at the household and branch levels could not be modeled. Siblings accounted for only 3.5% of the sample, and accounting for clustering by grade did not change the primary findings, but unaccounted correlation could have led to underestimated standard errors and overly narrow confidence intervals. Finally, numerous subgroup, interaction, and sensitivity analyses were performed; these were exploratory, and although the primary associations remained significant after FDR correction, the subgroup findings, including those for kindergarten children, were based on limited power.

## 5. Conclusions

In this private-school sample of children in Riyadh, higher screen time was associated with later bedtimes and non-restorative sleep, independent of age and sex, but not with lower physical activity. The association with sleep duration was largely explained by age. These cross-sectional associations do not establish causality, but they suggest that guidance on evening screen use and consistent school-night bedtimes, particularly from the early school years, merits evaluation in longitudinal and intervention studies that measure the timing and content of screen use.

## Figures and Tables

**Table 1:** Participant characteristics and lifestyle behaviors (*n* = 1208).

Variable	Category	*n*	%
**Age (years)**	Mean ± SD	9.1 ± 2.8	
	Range	4–15	
**Sex**	Female	694	57.5
	Male	514	42.5
**School level**	Kindergarten	176	14.6
	Elementary	735	60.8
	Middle	297	24.6
**BMI (kg/m^2^)**	Mean ± SD (*n* = 1203)	18.5 ± 4.6	
**Chronic health condition**	Any	192	15.9
	None	1016	84.1
**Daily screen time**	<1 h	244	20.2
	1–2 h	314	26.0
	2–3 h	337	27.9
	>3 h	313	25.9
**School-night bedtime**	Before 20:00	225	18.6
	20:00–21:00	766	63.4
	Around 22:00	170	14.1
	After 22:00	47	3.9
**Nightly sleep duration**	<7 h	114	9.4
	7–8 h	543	45.0
	9–10 h	492	40.7
	>10 h	59	4.9
**Waking refreshed**	Yes	904	74.8
	No	304	25.2
**Daily physical activity**	<30 min	583	48.3
	30–60 min	395	32.7
	1–2 h	165	13.7
	>2 h	65	5.4
**Sitting at school per day**	<2 h	5	0.4
	2–4 h	18	1.5
	4–6 h	267	22.1
	>6 h	918	76.0
**Regular sitting breaks**	Yes	774	64.1
	No	434	35.9

Values are *n* and % unless otherwise indicated. Percentages may not total 100% due to rounding. SD, standard deviation; BMI, body mass index, calculated from parent-reported height and weight (five implausible values set to missing). “Around 22:00” includes one free-text response of 21:00–21:30.

**Table 2:** Bedtime, sleep, and physical activity by daily screen time (*n* = 1208).

Outcome	<1 h (*n* = 244)	1–2 h (*n* = 314)	2–3 h (*n* = 337)	>3 h (*n* = 313)	χ^2^ (df); V	Gamma (95% CI)
Bedtime						
Before 20:00	69 (28.3)	85 (27.1)	37 (11.0)	34 (10.9)	132.31 (9); 0.191	0.403 (0.335–0.472)
20:00–21:00	157 (64.3)	203 (64.6)	230 (68.2)	176 (56.2)		
Around 22:00	18 (7.4)	24 (7.6)	56 (16.6)	72 (23.0)		
After 22:00	0 (0.0)	2 (0.6)	14 (4.2)	31 (9.9)		
Sleep duration						
<7 h	11 (4.5)	12 (3.8)	36 (10.7)	55 (17.6)	112.03 (9); 0.176	−0.336 (−0.403 to −0.269)
7–8 h	94 (38.5)	106 (33.8)	182 (54.0)	161 (51.4)		
9–10 h	121 (49.6)	172 (54.8)	112 (33.2)	87 (27.8)		
>10 h	18 (7.4)	24 (7.6)	7 (2.1)	10 (3.2)		
Waking refreshed						
No (non-restorative)	49 (20.1)	58 (18.5)	87 (25.8)	110 (35.1)	27.44 (3); 0.151	0.237 (0.142–0.333)
Yes	195 (79.9)	256 (81.5)	250 (74.2)	203 (64.9)		
Physical activity						
<30 min	121 (49.6)	137 (43.6)	151 (44.8)	174 (55.6)	28.63 (9); 0.089	−0.064 (−0.136 to 0.008)
30–60 min	74 (30.3)	113 (36.0)	129 (38.3)	79 (25.2)		
1–2 h	28 (11.5)	42 (13.4)	47 (13.9)	48 (15.3)		
>2 h	21 (8.6)	22 (7.0)	10 (3.0)	12 (3.8)		

Values are *n* (% within screen-time category). All chi-square tests *p* < 0.001. V, Cramér’s V. Gamma summarizes the ordered association between increasing screen time and later bedtime, longer sleep, not waking refreshed, and more physical activity, respectively; a CI including zero indicates no significant ordered association. CI, confidence interval; df, degrees of freedom.

**Table 3:** Adjusted associations between daily screen time and bedtime, sleep, and physical activity (*n* = 1208).

Screen Time	Late Bedtime ^a^	Sleep <7 h ^a^	Sleep ≥9 h ^a^	Non-Restorative Sleep ^a^	More Physical Activity ^b^
>3 h/day	Ref.	Ref.	Ref.	Ref.	Ref.
2–3 h/day	0.74 (0.51–1.08)	0.73 (0.46–1.17)	0.83 (0.58–1.18)	0.68 (0.48–0.96) *	1.04 (0.77–1.41)
1–2 h/day	0.37 (0.23–0.62) *	0.33 (0.17–0.66) *	1.81 (1.25–2.62) *	0.48 (0.32–0.72) *	0.88 (0.64–1.22)
<1 h/day	0.43 (0.24–0.78) *	0.49 (0.23–1.02)	1.12 (0.75–1.68)	0.56 (0.36–0.86) *	0.66 (0.46–0.95) *
Trend per higher category	1.43 (1.19–1.71) *	1.41 (1.12–1.78) *	0.88 (0.77–1.00)	1.26 (1.09–1.45) *	1.15 (1.02–1.29) *
*p* for non-linearity ^c^	0.246	0.214	<0.001	0.148	0.320
Age (per year)	1.35 (1.25–1.44) *	1.25 (1.15–1.37) *	0.74 (0.70–0.78) *	1.05 (0.99–1.11)	0.82 (0.79–0.86) *
Male sex	1.06 (0.77–1.47)	0.98 (0.65–1.48)	0.90 (0.70–1.16)	1.22 (0.93–1.59)	1.27 (1.02–1.58) *

Values are odds ratios (95% CI) adjusted for age and sex; * *p* < 0.05. ^a^ Binary logistic regression; late bedtime was defined as at or after 22:00, non-restorative sleep as not usually waking refreshed, and sleep ≥ 9 h was compared with ≤8 h. ^b^ Proportional odds (ordinal logistic) regression; OR > 1 indicates higher odds of a higher physical activity category. The trend OR is per one-category increase in screen time. ^c^ Likelihood ratio test comparing the categorical and linear-score models (2 df); a significant value indicates that the trend estimate does not adequately summarize the association. OR, odds ratio; CI, confidence interval.

**Table 4:** Sensitivity analyses for late bedtime and non-restorative sleep.

Model	*n*	2–3 h/Day	1–2 h/Day	<1 h/Day
**Late bedtime**				
Main model (age, sex)	1208	0.74 (0.51–1.08)	0.37 (0.23–0.62) *	0.43 (0.24–0.78) *
School level instead of age	1208	0.72 (0.49–1.05)	0.33 (0.20–0.55) *	0.36 (0.20–0.63) *
+ Chronic condition and BMI	1203	0.73 (0.50–1.06)	0.38 (0.23–0.63) *	0.44 (0.24–0.79) *
Excluding health conditions	1016	0.71 (0.47–1.06)	0.29 (0.17–0.51) *	0.30 (0.15–0.60) *
**Non-restorative sleep**				
Main model (age, sex)	1208	0.68 (0.48–0.96) *	0.48 (0.32–0.72) *	0.56 (0.36–0.86) *
School level instead of age	1208	0.67 (0.47–0.95) *	0.49 (0.33–0.73) *	0.56 (0.37–0.86) *
+ Chronic condition and BMI	1203	0.67 (0.48–0.95) *	0.48 (0.32–0.71) *	0.55 (0.36–0.86) *
Excluding health conditions	1016	0.61 (0.42–0.90) *	0.47 (0.31–0.72) *	0.50 (0.31–0.81) *
+ Bedtime	1208	0.70 (0.49–1.00) *	0.55 (0.37–0.82) *	0.62 (0.40–0.97) *

Values are odds ratios (95% CI) from binary logistic regression with screen time > 3 h/day as the reference; * *p* < 0.05. All models include sex. BMI, body mass index. “+ Bedtime” adds four-category school-night bedtime to the main model. CI, confidence interval.

## Data Availability

The de-identified data supporting the findings of this study are available from the corresponding author upon reasonable request.

## Supplementary Materials

The following supporting information can be downloaded at: https://www.mdpi.com/article/10.3390/jcm15218275/s1, Table S1: Assessment of the proportional odds assumption and linearity of the screen-time trend; Table S2: Benjamini–Hochberg false discovery rate correction; Table S3: Unadjusted and adjusted models and collapsed sleep-duration categories; Table S4: Simulated power in the kindergarten subgroup; Table S5: Sensitivity analysis accounting for clustering by school grade.

## Abbreviations

The following abbreviations are used in this manuscript:

BMI	Body Mass Index
CI	Confidence Interval
df	Degrees of Freedom
FDR	False Discovery Rate
LR	Likelihood Ratio
OR	Odds Ratio
SD	Standard Deviation
STROBE	Strengthening the Reporting of Observational Studies in Epidemiology

## Appendix A. Survey Items Analyzed in the Present Study (English Version)

Instructions: A parent or legal guardian completes this survey on behalf of their child. The survey was presented bilingually (Arabic and English); the English wording is shown below. The survey also included items not analyzed in the present study.

Section A1: Child Information

School level (kindergarten/elementary/middle school); grade; age (years); sex (male/female); height (cm); weight (kg); medical conditions (free text, or “None”).

Section A2: Sleep

Bedtime on school days: 6–7 pm (18:00–19:00)/8–9 pm (20:00–21:00)/10 pm (22:00)/after 10 pm (after 22:00).

Average hours of night sleep: <7 h/7–8 h/9–10 h/>10 h.

Does your child usually wake up feeling refreshed in the morning? Yes/No.

Section A3: Daily Activities

Time spent sitting at school (average per day): <2 h/2–4 h/4–6 h/>6 h.

Time spent on screens (TV, phone, computer) outside school: <1 h/1–2 h/2–3 h/>3 h.

Time spent in physical activity (sports, outdoor play, walking) per day: <30 min/30–60 min/1–2 h/>2 h.

Does your child regularly take breaks from sitting (every 30–60 min)? Yes/No.

Section A4: Consent

Confirmation of parent/guardian status and consent to participate (required to continue).
