# Supplementary material for: Associations of Screen Time with Bedtime, Sleep, and Physical Activity Among School-Aged Children in Riyadh: A Cross-Sectional Study
Source: J Clin Med. 2026 Oct 23;15(21):8275. doi: 10.3390/jcm15218275 (PMC13763923; doi:10.3390/jcm15218275)
Supplement: Supplementary file 1 [file jcm-15-08275-s001.zip › jcm-4616471-supplementary.pdf]

Supplementary Materials

Associations of Screen Time with Bedtime, Sleep, and Physical Activity Among School-Aged Children in Riyadh: A Cross-Sectional Study

Table S1. Assessment of the proportional odds assumption and of linearity of the screen-time trend.

Outcome / test	Statistic	p
**A. Brant test (model: screen-time score, age, sex)**		
Bedtime, 4 categories: omnibus	$\chi^2(6) = 15.17$	0.019
Screen time	$\chi^2(2) = 7.09$	0.029
Sleep duration, 4 categories: omnibus	$\chi^2(6) = 8.61$	0.197
Screen time	$\chi^2(2) = 4.02$	0.134
Sleep duration, collapsed to 3 categories: omnibus	$\chi^2(3) = 7.02$	0.071
Screen time	$\chi^2(1) = 3.23$	0.072
Physical activity, 4 categories: omnibus	$\chi^2(6) = 15.19$	0.019
Screen time	$\chi^2(2) = 5.28$	0.071
**B. Cumulative splits: OR (95% CI) per higher screen-time category**		
Sleep: ≥7 h vs. <7 h	0.71 (0.56–0.89)	0.004
Sleep: ≥9 h vs. ≤8 h	0.88 (0.77–1.00)	0.050
Sleep: >10 h vs. ≤10 h	0.98 (0.74–1.30)	0.910
Bedtime: 20:00 or later vs. before 20:00	1.17 (1.00–1.38)	0.052
Bedtime: ≥22:00 vs. earlier	1.43 (1.19–1.71)	<0.001
Bedtime: after 22:00 vs. earlier	2.23 (1.39–3.59)	<0.001
Physical activity: ≥30 min vs. <30 min	1.14 (1.01–1.29)	0.039
Physical activity: >60 min vs. ≤60 min	1.24 (1.06–1.45)	0.006
Physical activity: >2 h vs. ≤2 h	0.97 (0.74–1.26)	0.810
**C. Departure from linear trend (LR test, categorical vs. linear score, 2 df)**		
Late bedtime	$\chi^2(2) = 2.80$	0.246

Sleep <7 h	$\chi^2(2) = 3.08$	0.214
Sleep ≥9 h	$\chi^2(2) = 17.74$	<0.001
Non-restorative sleep	$\chi^2(2) = 3.82$	0.148
Physical activity (proportional odds)	$\chi^2(2) = 2.28$	0.320

All models adjusted for age and sex (n = 1208). In panel B, each OR is the odds of being above the cut-point per one-category increase in screen time. A significant Brant test indicates that the proportional odds assumption is violated.

Table S2. Benjamini–Hochberg false discovery rate (FDR) correction.

**Test**	**p**	**q (FDR)**
**A. Screen-time trend, primary outcomes (protocol)**		
Sleep <7 h	0.004	0.004
Non-restorative sleep	0.001	0.002
**B. Screen-time trend, all outcomes (primary and secondary)**		
Late bedtime	<0.001	<0.001
Sleep <7 h	0.004	0.007
Sleep ≥9 h	0.050	0.060
Sleep >10 h	0.910	0.910
Non-restorative sleep	0.001	0.004
Physical activity	0.018	0.026
**C. Category-specific contrasts in Table 3 (vs. >3 h/day; 15 tests)**		
Late bedtime, 2–3 h/day	0.119	0.179
Late bedtime, 1–2 h/day	<0.001	0.002
Late bedtime, <1 h/day	0.006	0.017
Sleep <7 h, 2–3 h/day	0.197	0.268
Sleep <7 h, 1–2 h/day	0.002	0.006
Sleep <7 h, <1 h/day	0.058	0.097
Sleep ≥9 h, 2–3 h/day	0.293	0.366
Sleep ≥9 h, 1–2 h/day	0.002	0.006

Sleep ≥9 h, <1 h/day	0.567	0.607
Non-restorative sleep, 2–3 h/day	0.030	0.055
Non-restorative sleep, 1–2 h/day	<0.001	0.002
Non-restorative sleep, <1 h/day	0.008	0.021
Physical activity, 2–3 h/day	0.797	0.797
Physical activity, 1–2 h/day	0.456	0.526
Physical activity, <1 h/day	0.025	0.053

Models adjusted for age and sex (n = 1208). q < 0.05 indicates significance after FDR correction.

Table S3. Unadjusted and adjusted models and collapsed sleep-duration categories.

**Outcome**	**2–3 h/day**	**1–2 h/day**	**<1 h/day**	**Trend**
**A. Adjusted for age, sex, BMI, and chronic health condition (n = 1203)**				
Late bedtime	0.73 (0.50–1.06)	0.38 (0.23–0.63)	0.44 (0.24–0.79)	1.42 (1.19–1.70)
Sleep <7 h	0.75 (0.47–1.20)	0.34 (0.17–0.68)	0.50 (0.24–1.05)	1.40 (1.11–1.76)
Sleep ≥9 h	0.83 (0.58–1.19)	1.80 (1.24–2.61)	1.11 (0.74–1.65)	0.89 (0.78–1.01)
Non-restorative sleep	0.67 (0.48–0.95)	0.48 (0.32–0.71)	0.55 (0.36–0.86)	1.26 (1.09–1.45)
Physical activity (ordinal)	1.04 (0.77–1.40)	0.87 (0.63–1.20)	0.66 (0.46–0.94)	1.15 (1.03–1.29)
**B. Sleep duration collapsed to <7, 7–10, and >10 h (proportional odds; n = 1208)**				
Longer sleep category	1.09 (0.71–1.68)	2.15 (1.28–3.61)	1.65 (0.93–2.93)	0.80 (0.67–0.96)
**C. Unadjusted vs. adjusted for age and sex (binary logistic regression; n = 1208)**				
Late bedtime, unadjusted	0.53 (0.38–0.76)	0.18 (0.12–0.29)	0.16 (0.10–0.28)	2.01 (1.71–2.35)
Late bedtime, adjusted	0.74 (0.51–1.08)	0.37 (0.23–0.62)	0.43 (0.24–0.78)	1.43 (1.19–1.71)
Sleep <7 h, unadjusted	0.56 (0.36–0.88)	0.19 (0.10–0.36)	0.22 (0.11–0.43)	1.86 (1.51–2.29)
Sleep <7 h, adjusted	0.73 (0.46–1.17)	0.33 (0.17–0.66)	0.49 (0.23–1.02)	1.41 (1.12–1.78)
Sleep ≥9 h,	1.22 (0.88–1.69)	3.70 (2.66–5.15)	2.95 (2.08–4.18)	0.63 (0.57–0.71)

unadjusted				
Sleep ≥9 h, adjusted	0.83 (0.58–1.18)	1.81 (1.25–2.62)	1.12 (0.75–1.68)	0.88 (0.77–1.00)
Non-restorative sleep, unadjusted	0.64 (0.46–0.90)	0.42 (0.29–0.60)	0.46 (0.31–0.68)	1.35 (1.19–1.53)
Non-restorative sleep, adjusted	0.68 (0.48–0.96)	0.48 (0.32–0.72)	0.56 (0.36–0.86)	1.26 (1.09–1.45)

Values are odds ratios (95% CI) with screen time >3 h/day as the reference. Trend ORs are per one-category increase in screen time. BMI, body mass index.

Table S4. Simulated power in the kindergarten subgroup (n = 176).

**Outcome**	**Events (%)**	**Assumed trend OR**	**Power**
Late bedtime	12 (6.8)	1.48 (as in elementary school)	0.28
		1.50	0.28
		2.00	0.69
Non-restorative sleep	26 (14.8)	1.25 (as in elementary school)	0.17
		1.50	0.50
		2.00	0.92

Power was estimated from 1000 simulated datasets using the observed kindergarten screen-time distribution and outcome prevalence, with a two-sided logistic trend test at $\alpha = 0.05$. Observed kindergarten estimates (adjusted for age and sex): late bedtime trend OR = 1.68 (95% CI 0.94–3.01); non-restorative sleep trend OR = 0.92 (95% CI 0.57–1.46).

Table S5. Sensitivity analysis accounting for clustering by school grade.

**Outcome**	**Model-based OR (95% CI)**	**p**	**Grade-clustered OR (95% CI)**	**p**
Late bedtime	1.43 (1.19–1.71)	<0.001	1.43 (1.09–1.88)	0.017
Sleep <7 h	1.41 (1.12–1.78)	0.004	1.41 (1.17–1.71)	0.003
Sleep ≥9 h	0.88 (0.77–1.00)	0.050	0.88 (0.76–1.01)	0.072
Non-restorative sleep	1.26 (1.09–1.45)	0.001	1.26 (1.04–1.53)	0.026

Physical activity (ordinal)	1.15 (1.02–1.29)	0.018	1.15 (0.99–1.33)	0.058

Values are odds ratios per one-category increase in screen time, adjusted for age and sex (n = 1208). Point estimates are identical in both columns; grade-clustered estimates use cluster-robust standard errors with school grade as the cluster (10 grades, kindergarten level 3 to grade 9), and confidence intervals and p values are based on the t distribution with 9 degrees of freedom to account for the small number of clusters. Household and school branch identifiers were not retained in the analysed dataset; 42 children (3.5%) were siblings from 21 households.
